# pH‐Responsive Hexa‐Histidine Metal Assembly (HmA) with Enhanced Biocatalytic Cascades as the Vehicle for Glucose‐Mediated Long‐Acting Insulin Delivery

**DOI:** 10.1002/advs.202301771

**Published:** 2023-06-02

**Authors:** Sijie Zhou, Ruhui Yang, Xiaoling Xie, Liwen Wang, Shengwu Zheng, Na Li, Sicheng Tang, Xingjie Zan

**Affiliations:** ^1^ School of Ophthalmology and Optometry Eye Hospital School of Biomedical Engineering Wenzhou Medical University Wenzhou 325035 China; ^2^ Wenzhou Institute Wenzhou Key Laboratory of Perioperative Medicine University of Chinese Academy of Sciences Wenzhou 325001 China; ^3^ Department of Ophthalmology Huzhou Central Hospital Affiliated Central hospital Huzhou University Huzhou 313000 China; ^4^ Wenzhou Celecare Medical Instruments Co., Ltd Wenzhou 325000 China

**Keywords:** diabetes, glucose‐response, hexahistidine‐metal‐assembly, insulin delivery, pH sensitive

## Abstract

Diabetes has been listed as one of the three major diseases that endanger human health. Accurately injecting insulin (Ins) depending on the level of blood glucose (LBG) is the standard treatment, especially controlling LBG in the long‐term by a single injection. Herein, the pH‐responsive hexa‐histidine metal assembly (HmA) encapsulated with enzymes (GOx and CAT) and Ins (HmA@GCI) is engineered as the vehicle for glucose‐mediated insulin delivery. HmA not only shows high proteins loading efficiency, but also well retained proteins activity and protect proteins from protease damage. Within HmA, the biocatalytic activities of enzymes and the efficiency of the cascade reaction between GOx and CAT are enhanced, leading to a super response to the change of LBG with insulin release and efficient clearance of harmful byproducts of GOx (H_2_O_2_). In the treatment of diabetic mice, HmA@GCI reduces LBG to normal in half an hour and maintains for more than 5 days by a single subcutaneous injection, and nearly 24 days with four consecutive injections. During the test period, no symptoms of hypoglycemia and toxicity to tissues and organs are observed. These results indicate that HmA@GCI is a safe and long‐acting hypoglycemic agent with prospective clinical application.

## Graphical abstract

In this work, HmA@GOx@CAT@Ins (HmA@GCI) nanoparticles are prepared under mild conditions. The nanoparticles have high proteins loading capacity and protect proteins from protease. More importantly, HmA@GCI can improve enzyme activity and enhance the efficiency of cascade reaction, and have long‐term hypoglycemic effect in vivo, providing a safe and long‐acting hypoglycemic agent with prospective clinical application.

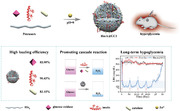

## 1 Introduction

Diabetes, a chronic disease with the major pathological feature of raising blood glucose, can give rise to serious damage to many body systems when it is in an uncontrolled state over periods, particularly the nerves and blood vessels.^[^ ^1^ ^]^ According to reports by the International Diabetes Federation, approximately 536.6 million adults live with diabetes worldwide in 2021, and it is projected to reach 783.2 million by 2045.^[^ ^2^ ^]^ Diabetes is listed as one of the three major diseases that endanger human health (the other two are cancer and cardiovascular disease).^[^ ^3^ ^]^ In clinical treatment for diabetes, the direct injection of insulin (Ins) or Ins analogs, a peptide hormone to reduce the level of blood glucose (LBG) by promoting the metabolism of glucose in liver, fat, and skeletal muscle cells, is a popular and standard method.^[^ ^4^ ^]^ Although various devices, including Ins syringes and Ins pens, have been developed for ease of patients' self‐injection of Ins, the daily requirement on injection in the long term brings great pain and inconvenience, which leads to low compliance for most diabetics.^[^ ^5^, ^6^ ^]^ More seriously, minimal control over the dosage of injected Ins by patients, overdosage or inadequate injection, results in unexpected therapeutic outcomes.^[^ ^7^, ^8^ ^]^ Therefore, how to ensure the timely control of blood glucose and the long‐term release of Ins is still the focus of research.

In healthy people, the body can continuously secrete insulin according to the LBG.^[^ ^9^ ^]^ However, for patients with type 1 diabetes or advanced type 2 diabetes, the body cannot secrete enough Ins for use. Thus, simulating the function of the human pancreas by injecting a glucose‐responsive delivery vehicle for Ins could be a way to adapt the LBG accurately in realtime and safely achieve individualized treatment of euglycemia in the long‐term, as offered by many researchers from different disciplines. In the past decades, to receive the long‐acting release of Ins, major efforts and advances have been made to refine Ins formulations,^[^ ^10^, ^11^ ^]^ including construction of Ins analogues,^[^ ^12^ ^]^ chemically modified long‐acting insulin^[^ ^13^ ^]^ and self‐assembled Ins repositories.^[^ ^14^, ^15^ ^]^ The strategy of combining glucose‐sensitive elements with self‐assembled Ins repositories enables Ins to achieve long‐term sustained release through glucose responsive release.^[^ ^16^, ^17^ ^]^ At present, various glucose‐responsive vehicles have been developed, including glucose‐binding protein‐based,^[^ ^18^, ^19^, ^20^ ^]^ phenylboronic acid (PBA)‐based,^[^ ^21^, ^22^, ^23^, ^24^ ^]^ and glucose oxidase (GOx)‐based^[^ ^14^, ^16^, ^25^, ^26^ ^]^ systems. Among these systems, the GOx (glucose‐specific enzyme that catalyzes glucose to gluconic acid and H_2_O_2_ in the presence of oxygen) based system has received special attention due to its super specificity to glucose and high sensitivity to the LBG. In GOx‐based systems, GOx and Ins were coloaded into pH‐sensitive carriers. In the presence of glucose, the local pH is reduced due to the gluconic acid produced under the catalysis of GOx, which induces the shrinkage, swelling, degradation or disintegration of the pH‐sensitive carriers,^[^ ^15^, ^16^, ^27^, ^28^ ^]^ thus releasing Ins. Since the rate of producing gluconic acid in this reaction was dependent on the concentration of glucose, the releasing dosage of Ins could be controllable by the LBG: fast release under high blood glucose levels and slow or no release under normal blood glucose levels, promoting the retention of Ins in repositories. There have been numerous pH‐sensitive materials (synthetic macromolecules, small molecular peptides, naturally derived‐polymers, polysaccharides, proteins, etc.) with different formulations, such as hydrogels,^[^ ^15^ ^]^ vesicles,^[^ ^29^, ^30^, ^31^ ^]^ and micro‐nano‐particles,^[^ ^16^, ^32^ ^]^ have been demonstrated to hunt the goal of stimulating human pancreas.

However, there are many unavoidable issues in GOx‐based glucose responsiveness. The first is the byproduct H_2_O_2,_ which is damaged to cells by causing lipid, protein denaturation or DNA damage, leading to serious inflammation in biological tissues and organs.^[^ ^33^ ^]^ To avoid the damage of H_2_O_2_, catalase (CAT) or CAT‐like inorganic nanoparticles had to be introduced into the above pH‐sensitive materials, in which CAT or CAT‐like inorganic nanoparticles catalyze the decomposition of hydrogen peroxide to nontoxic products oxygen and water, maintaining redox equilibrium in the body.^[^ ^27^, ^34^, ^35^, ^36^ ^]^ Secondly, how to properly store the bioactivity of encapsulated proteins (GOX, CAT, and Ins) and release as needed under physiological conditions. Once injected, the carriers are surrounded by body fluid buffer, enzymes that deactivate the loaded protein, and immune cells that are responsible for cleaning foreign materials. In addition, as proteins, their quarternary structures are critical to their activities but are very fragile to the microenvironmental changes during the loading and delivery process. Therefore, achieving a rapid response to the change in the LBG and extending the release of Ins with the desired kinetics in one injection is challenging, including proper preservation for the bioactivity of encapsulated proteins, avoiding attack from immune cells, high sensitivity to the change in the LBG, fast pH change and Ins release, high payload of required cargoes, low biological toxicity or degradation ability of carriers, etc., especially in long term usage.

**Scheme 1:**
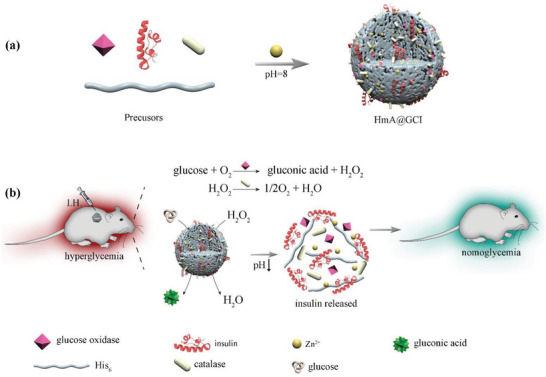
Schematically illustration of a) co‐encapsulating GOx, CAT, and Ins into HmA and b) rapid response to glucose and extended release of Ins in vivo for T1D treatment.

Previously, we reported a nanoparticle (HmA) based on the coordinative interaction between hexehistidine (His_6_) and metal ion (Zn^2+^),^[^ ^37^ ^]^ into which various proteins and peptides were encapsulated during the formation of HmA through co‐assembly process.^[^ ^38^, ^39^ ^]^ Most importantly, HmA could store the bioactivity of encapsulated protein properly, protect them from enzyme degradation, and release the proteins in the decreased pH environment so that the encapsulated proteins completely performed their biofunctions in vitro and in vivo.^[^ ^40^ ^]^ Considering these features of HmA and the above concerns, we presented a glucose‐responsive Ins delivery system by co‐encapsulating GOx, CAT, and Ins into HmA (HmA@GCI) (**Scheme** 1a), which displayed a high payload of encapsulated proteins (GOx, CAT, and Ins), and enhanced biocatalytic activities of GOx and CAT. When HmA@GCI was injected subcutaneously into T1D mice, glucose was catalyzed into gluconic acid and H_2_O_2_ (Scheme 1b). With the generation of gluconic acid, the local pH decreased and led to the disintegration of HmA particles and Ins release. At the same time, the toxic byproduct H_2_O_2_ was catalyzed by CAT to oxygen and water (Scheme 1b). Promoted efficiency of the biocatalytic cascade reaction between GOx and CAT reduced the damage of byproduct of GOx in the sensitive process to glucose. Our data demonstrated that this strategy can maintain a euglycemia state for more than 5 days by a single subcutaneous injection and have excellent hypoglycemic effect and biocompatibility with undetected immunogenicity and systematically biological toxicity after continuous injection for more than 24 days. It is expected to become a long‐term intelligent carrier for glucose‐mediated insulin transdermal administration in the treatment of T1D.

## 2 Results and Discussion

### 2.1 Characterization of Nanoparticles

**Figure 1:**
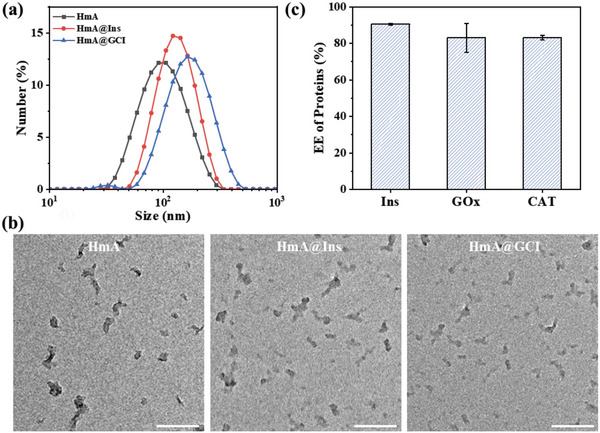
a) Hydrodynamic size distribution, and b) TEM images of HmA, HmA@Ins, and HmA@GCI. Scale bar, 200 nm. c) The entrapment efficiency (EE%) of Ins, GOx, and CAT into HmA.

The size distribution and zeta potential of synthesized nanoparticles, HmA, HmA@Ins, and HmA@GCI, were determined using dynamic light scattering and are presented in **Figure** **1a** and **Table** **1**, The results showed that all three types of nanoparticles, whose sizes were 99.60, 117.95, and 167.1 nm, respectively, were nanoscale with a narrow size distribution (PDI < 0.2). Additionally, the zeta potentials of all nanoparticles were between 18.2 to 23.6 mV. TEM analysis (Figure 1b) revealed that all nanoparticles had irregular shapes with sizes smaller than those measured by dynamic light scattering, which could be attributed to the dehydration process during sample preparation. Further analysis demonstrated that the entrapment efficiency (EE%) of Ins, GOx, and CAT in HmA@GCI was 90.43%, 83.08%, and 83.1%, respectively (Figure 1c), as calculated from the standard curve in Figure S1 (Supporting Information). Notably, the EE% of Ins by HmA was significantly higher than previously reported glucose‐responsive Ins delivery systems: Wu et al. reported an EE% range of 67–75%,^[^ ^41^ ^]^ whereas Guo et al. found an EE% of 60%.^[^ ^42^ ^]^ According to the previously reported work^[^ ^39^, ^43^ ^]^, the high encapsulation efficiency (EE%) of HmA can be attributed to the different interaction modes of the drugs with His_6_ or Zn^2+^. Firstly, at neutral and alkaline pH values, Ins, GOx, and CAT are negatively charged and have electrostatic interactions with positively charged HmA. Secondly, Ins, GOx, and CAT are proteins, and their amino acid residues, such as carboxyl group, imidazole group, and amino group, can have metal coordination interactions with Zn^2+^. The above reasons can make HmA have high EE% of Ins, GOx, and CAT. (Table **1**)

**Table 1:** The size, PDI, and zeta potential of HmA, HmA@Ins, and HmA@GCI

Nanoparticles	Size [nm]	PDI	Zeta‐potential [mV]
HmA	99.60	0.176	23.6
HmA@Ins	117.95	0.115	18.2
HmA@GCI	167.1	0.131	19.8

### 2.2 Glucose Responsiveness of HmA@GCI

**Figure 2:**
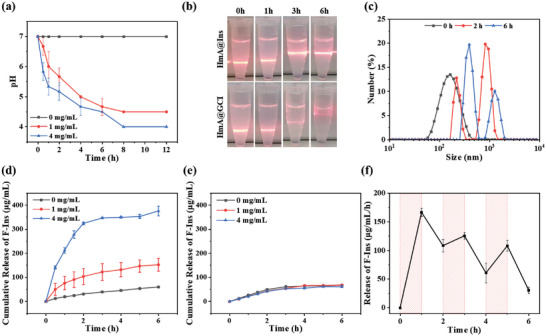
a) pH changes over time of HmA@GCI solution incubated with various concentrations of glucose. b) Disintegration over incubated time of HmA@Ins (top) and HmA@GCI (bottom) in glucose (4 mg mL^−1^). c) Hydrodynamic size distribution of HmA@GCI incubated with glucose solution (4 mg mL^−1^) at various time. Cumulative insulin release from HmA@GCI (d) and HmA@Ins (e) at various concentrations of glucose. f) Pulsatile release profile of FITC‐Ins when exposed between a hyperglycemic and normal state alternatively.

The glucose‐responsive release of Ins is based on the pH‐responsive disintegration of HmA and the glucose specificity of GOx. The oxidation of glucose by GOx would generate gluconic acid, resulting in a decrease of local pH. Such an acidic environment would initiate the disassembly of HmA. To validate the glucose responsive process of the particles, HmA@GCI were incubated under different glucose concentrations (0, 1, and 4 mg mL^−1^) and the pH was monitored over time, with the HmA@Ins served as a control. While the pH value of in the suspension of HmA and HmA@Ins remained unchanged (Figure S2, Supporting Information), the pH of HmA@GCI exhibited a significant glucose concentration‐ and incubation time‐dependence (**Figure** **2a**). The pH values decreased gradually with increasing incubation time to 4 for 4 mg mL^−1^ glucose and 4.5 for 1 mg mL^−1^, indicating that the glucose was oxidized to gluconic acid under the catalysis of GOx. During this period, the disintegration of the particles could be observed by the naked eye. Taking the HmA@GCI in the solution of 4 mg mL^−1^ glucose as an example, the Tyndall effect gradually weakened and finally disappeared (Figure 2b), which suggests the disintegration of the nanoparticles. Such a disintegration process could be further supported by dynamic light scattering. In contrast to HmA and HmA@Ins (Figure S3, Supporting Information), multiple peaks were observed in the particle size distrubution of HmA@GCI (Figure 2c), which could be attributed to a disintegration and aggregation mechanism, as demonstrated by previous literatures.^[^ ^16^ ^]^

### 2.3 In Vitro Release of Ins from Nanoparticles

To further validate the glucose responsive release of HmA@GCI, the Ins release curves of HmA@GCI at different glucose concentrations were measured. As shown in Figure 2d, the F‐Ins release were consistent with the trends of pH change (Figure 2a), depending on both glucose concentration and incubation time. The F‐Ins was continuously released within incubation time and exhibited a higher release rate at elevated glucose levels. At the end of the tested period, the cumulative release of Ins was approximately 375 µg mL^−1^ (96.6%) at a glucose concentration of 4 mg mL^−1^ (hyperglycemia level), while only approximately 152 µg mL^−1^ (39.2%) at a glucose concentration of 1 mg mL^−1^ (normal blood glucose level) and close to 60 µg mL^−1^ (15.5%) at a glucose concentration of 0 mg mL^−1^. However, no significant difference was observed in F‐Ins released from HmA@Ins under different concentrations of glucose, with a total release of approximately 65 µg mL^−1^ (16.25%) by the end of testing (Figure 2e). Notably, there was a rapid initial F‐Ins release within the first 2 h at a glucose concentration of 4 mg mL^−1^, accounting for approximately 323 µg mL^−1^ (83.2%), which could have potential implications for rapid blood glucose control in vivo.

To simulate the fluctuation of blood glucose levels in the daily life of diabetics, we alternately used hyperglycemia and normal blood glucose every hour. As shown in Figure 2f, the resulting F‐Ins release curve displayed pulsatile behavior, signifying that insulin release increased with elevations in glucose concentration, while decreasing when glucose levels dropped. This demonstrates the capability of HmA@GCI to intelligently respond to changes in blood glucose levels, with rapid release observed during high glucose levels and slower release at normal levels. Consequently, this system enables “on‐off” release of insulin, providing potential advantages for diabetes treatment including the ability to promptly control blood glucose within the normal range, thus preventing diabetic hyperosmotic coma caused by high glucose levels. Moreover, glucose‐dependent release facilitates accurate insulin dosing according to blood glucose levels, potentially avoiding the occurrence of hypoglycemia.

### 2.4 Bioactivities and Biocatalytic Cascades of HmA@GCI

It is well known that the secondary structure of proteins plays a key role in maintaining their activity. Thus, the secondary structures of Ins, Gox, and CAT before encapsulation (native) and after release were analyzed. As shown in Figure S4 (Supporting Information), no significant discrepancy between the released proteins (Ins, GOx, and CAT) from HmA and the native proteins was observed, which was further supported by the secondary structure proportion analysis (Figure S5, Supporting Information).

**Figure 3:**
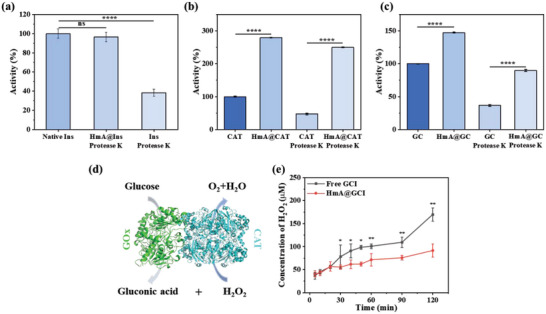
Bioactivity assays of a) HmA@Ins, b) HmA@CAT, and c) HmA@GC and their native counterparts under protease K treatment or control conditions (no treatment of protease K). d) The scheme of biocatalytic cascades of GOx and CAT. e) The biocatalytic cascades of free GCI and HmA@GCI.

The existence of proteases in the human environment creates a challenge for protein drug delivery due to susceptibility to inactivation. To assess the protective effect of HmA on exogenous Ins, GOx, and CAT, HmA@Ins, HmA@GOx, HmA@CAT, and HmA@GC were co‐incubated with protease K to measure the activities of the proteins. As shown in **Figure** **3**a–c and Figure S6a (Supporting Information) (HmA@GOx and its native counterparts), HmA@Ins, HmA@GOx, HmA@CAT, and HmA@GC were not affected by protease K compared to free proteins, which showed a significant reduction in enzyme activity. This result could be further supported by the data in Figure S6b (Supporting Information) of visual color change in the FOX solution after mixing the solution of H_2_O_2_ with free catalase and HmA@CAT with/without protease K with reaction time and their corresponding reaction kinetics on degrading H_2_O_2_ (Figure S6c, Supporting Information). It is worth mentioning that HmA@CAT was found to be 2.79 times more efficient in removing hydrogen peroxide than free CAT, while the activity of HmA@GC was 1.4 times higher than that of free GC. These results indicated that the encapsulation process did not affect the activity of proteins, and HmA had the ability to protect enzymes from protease K degradation, and could enhance the activity of some enzymes, such as CAT.

GOx and CAT were co‐encapsulated into HmA nanoparticles (HmA@GC). As shown in Figure 3d, under an aerobic environment, glucose was catalyzed by GOx to gluconic acid and H_2_O_2_, followed by the catalysis of H_2_O_2_ to H_2_O and O_2_ by CAT. To simulate the glucose‐triggered enzyme cascade reaction in vivo, free GCI solution and HmA@GCI nanoparticles were incubated with high glucose solution (4 mg mL^−1^) at 37 °C, and the content of H_2_O_2_ in the solution was determined. As shown in Figure 3e, there was no significant difference in the content of H_2_O_2_ between free GC and HmA@GCI within 10 min. However, after 10 min, the content of H_2_O_2_ in HmA@GCI solution was significantly lower than that in free GC. At 120 min, the content of H_2_O_2_ in free GCI group was 1.85 times higher than that in HmA@GCI group. It can be seen that the cascade reaction efficiency of GOx and CAT was improved after HmA encapsulation, which laid a foundation for rapid clearance of H_2_O_2_ in vivo.

### 2.5 Biocompatibility of Nanoparticles In Vitro

**Figure 4:**
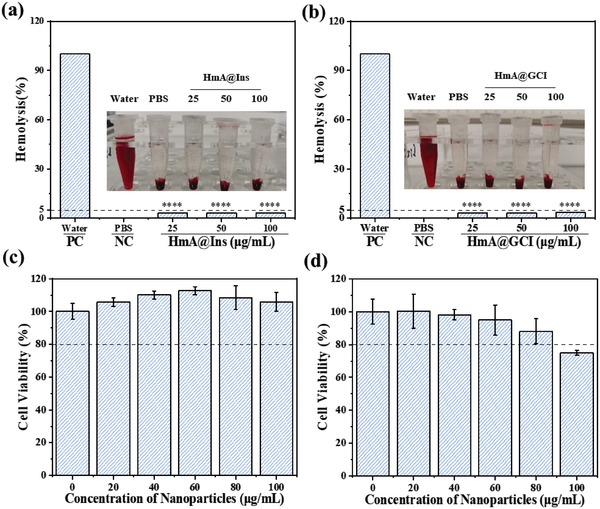
Hemolysis test of red blood cells at various concentrations of a) HmA@Ins and b) HmA@GCI for 4 h. c) Cell viability of dendritic cells (DCs) and d) NIH3T3 cells after co‐incubation with HmA at various concentrations for 24 h.

Before in vivo experiments, the biocompatibility of the nanoparticles was assessed through hemolysis and cytotoxicity experiments in vitro. As shown in **Figure** **4a‐b**, the hemolysis test revealed that HmA@Ins and HmA@GCI did not cause any significant hemolysis (less than 5%) even at high particle concentrations (up to 100 µg mL^−1^) after 4 h of incubation, as compared to the positive control (water). When HmA was incubated with dendritic cells (DCs) and NIH3T3 cells for 24 h, HmA showed no cytotoxicity toward DCs but exhibited slight cytotoxicity toward NIH3T3 cells at a concentration higher than 80 µg mL^−1^ (Figure 4c‐d). These results showed that these nanoparticles have good biocompatibility and are suitable for follow‐up experimental studies in vivo.

### 2.6 In Vivo Retention of Ins and Antidiabetic Studies of HmA@GCI in T1D Mice

**Figure 5:**
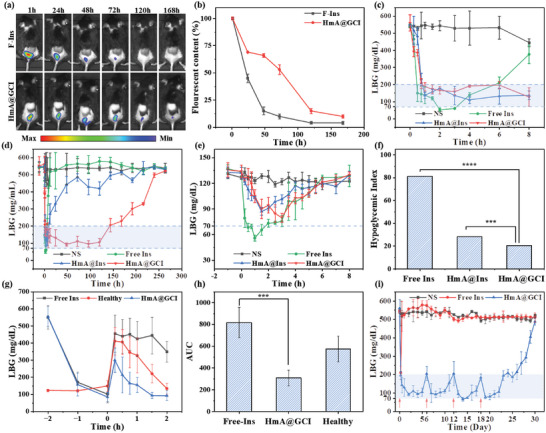
a) The retention of F‐Ins and HmA@GCI in vivo. b) Corresponding fluorescent content of injection site over time after injection. c) Level of blood glucose (LBG) in diabetic mice within the first 8 h. LBG in d) diabetic mice and e) healthy mice after subcutaneous injection with NS, free Ins, HmA@Ins, and HmA@GCI. f) Hypoglycemia index in different groups. g) Glucose tolerance test in healthy and diabetic mice. h) The responsiveness to intraperitoneal glucose tolerance test (IPGTT) was calculated based on the area under the curve from 0 to 2 h. i) LBG in diabetic mice after continuous injection with HmA@GCI.

To evaluate the retention of Ins in vivo, F‐Ins and HmA@GCI were subcutaneously injected into STZ‐induced T1D mice, which were imaged under MOIS and shown in **Figure** **5**a. The fluorescent signal of free F‐Ins disappeared much more rapidly and was hardly observed after 3 days, compared to that of HmA@GCI. To be specific, the total fluorescence of free F‐Ins decreased from 100% to approximately 15% within 2 days and further declined to 0% after 3 days, as shown in Figure 5b. In contrast, more than 50% of total fluorescence of HmA@GCI was retained after 3 days, and approximately 10% remained even at 7 days, suggesting a slower retention rate.

To evaluate the efficacy of HmA@GCI in the treatment of diabetes, T1D mice induced by STZ (Figure S7, Supporting Information) were divided into four groups and subcutaneously injected with normal saline, free Ins, HmA@Ins, or HmA@GCI. The LBG in each group was closely monitored at the preset time. As shown in Figure 5c, while free Ins had a rapid hypoglycemic effect, it also carried a high risk of inducing hypoglycemia (less than 70 mg dL^−1^) within 2 h. In addition, the LBG returned to hyperglycemia (higher than 200 mg dL^−1^) after 6 h. For the group treated with HmA@Ins, the LBG reached normal blood glucose after approximately one hour, was maintained for approximately 8 h, and then became hyperglycemia after 12 h. The hypoglycemic effect could be ascribed to the burst release of the Ins adhered to the surface of HmA. The injection of HmA@GCI could also quickly reduce the LBG to normal in half an hour, and was maintained for almost 5 days (Figure 5d). Compared with the work of Wang et al.,^[^ ^44^ ^]^ HmA@GCI had a longer hypoglycemic effect, which could be attributed to the longer retention time in vivo. In addition, to clarify the risk of hypoglycemia caused by HmA@GCI, injection of normal saline, free Ins, HmA@Ins, and HmA@GCI into healthy mice was performed. The LBG was monitored at the desired time points (Figure 5e), based on which the hypoglycemic index (low value suggests a low risk of hypoglycemia) was calculated and is shown in Figure 5f. The hypoglycemic index of mice treated with free Ins was significantly higher than that of mice treated with HmA@GCI, indicating a very low risk of hypoglycemia of HmA@GCI. These results shows that encapsulating Ins into HmA can reduce the risk of hypoglycemia, suggesting that the HmA has the ability to control the release of Ins. Whereas HmA@Ins was unable to maintain normal blood glucose levels beyond 8 h, HmA@GCI, which is endowed with intelligent glucose response characteristics through the introduction of GOx and pH sensitivity of HmA demonstrated significant therapeutic effects.

In addition, glucose tolerance experiments were carried out to explore the dynamic changes of different preparations of insulin in vivo. After the diabetic mice were subcutaneously injected with free insulin and HmA@GCI respectively, the healthy mice were subcutaneously injected with normal saline as the control. When the blood glucose of the mice decreased to a normal level, the mice were given intraperitoneal injection of glucose (2 g kg^−1^). As shown in the Figure 5g, the blood glucose of the mice increased rapidly to above 300mg dL^−1^ in 15 min. Then, those injected with HmA@GCI decreased below the normal level within 1.5 h. Whereas those injected with free insulin remained high (about 400 mg dL^−1^). Area under curve (AUC) (Figure 5h) showed that mice injected with HmA@GCI showed increased sensitivity to intraperitoneal glucose tolerance test (IPGTT) similar to that of healthy mice. These results confirm that HmA@GCI has a good glucose response in vivo and can quickly respond to the changes in blood glucose so as to release insulin in time. This characteristic offers convenience to diabetes and eliminates the need for administering insulin before meals to prevent postprandial hyperglycemia.

To confirm the stable and safe hypoglycemic effect induced by HmA@GCI, we repeatedly injected HmA@GCI into diabetic mice for four times. As demonstrated in the Figure 5i, a single injection could maintain a normal blood glucose state for about 5 days. When the blood glucose rebounded on the 6^th^ day, HmA@GCI was injected repeatedly and the blood glucose decreased to normal without any significant risk of hypoglycemia in the whole process. Thus, it can be seen that the application of the long‐acting preparation can significantly reduce the number of injections, from traditional multiple daily doses to a single dose over multiple days. This approach can not only effectively improve the compliance of diabetes, but also reduce the risk of atrophy and infection at the injection site caused by repeated injection. Therefore, as an insulin repository and glucose responsive release medium, HmA@GCI provides a new strategy for long‐term treatment of diabetes.

### 2.7 In Vivo Biosafety Assessment

**Figure 6:**
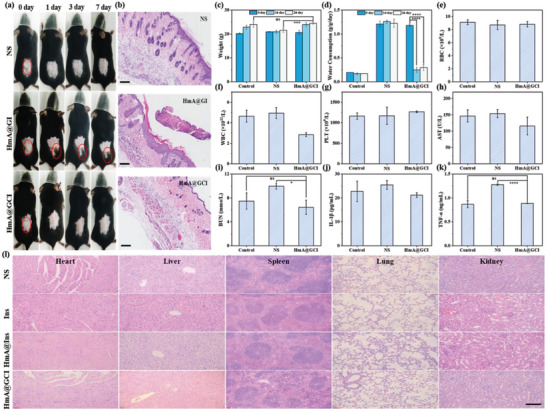
a) Pictures of mice dorsum treated with subcutaneous injection of NS, HmA@GI, or HmA@GCI after different days. Red circles refer to injection sites. b) H&E stained sections of the subcutaneous injection site with surrounding tissues. Scale bar, 200 µm. c) The weight change of healthy mice and diabetic mice injected with NS and HmA@GCI. d) The water consumption of healthy mice and diabetic mice injected with NS and HmA@GCI. e–g) The blood routine examination of RBC, WBC, and PLT of healthy mice and diabetic mice injected with NS and HmA@GCI at the 24^th^ day. Blood biochemistry levels including h) AST, i) BUN after treatment at the 24^th^ day. j) Serum levels of interleukin‐1*β* (IL‐1*β*) and tumor necrosis factor‐*α* (TNF‐*α*) in healthy mice and diabetic mice after injection with NS and HmA@GCI. l) H&E stained section of major organs after injected with NS, Ins, HmA@Ins, and HmA@GCI at the 24^th^ day, respectively. Scale bar, 100 µm.

To evaluate the biological safety of Ins, HmA@Ins, and HmA@GCI, mice were subcutaneously injected with normal saline, Ins, HmA@Ins, HmA@GI, and HmA@GCI. On days 1, 3, and 7, the injection sites of the mice were photographed. Seven days later, all mice were euthanized, and their skins were taken for H&E staining. As shown in **Figure** **6**a, the skin of mice injected with HmA@GI showed obvious skin ulceration on the first day, while the skin of mice injected with HmA@GCI remained intact. From the skin tissue section (Figure 6b), obvious skin defects could be seen in the HmA@GI group, but there were no obvious defects in the control group and HmA@GCI group. Thus, it is consistent with the results of scavenging hydrogen peroxide in vitro that CAT encapsulated in HmA can remove H_2_O_2_ in time and avoid inflammatory damage.

To confirm the potential clinical application of nanoparticles, we conducted in vivo long‐term biosafety evaluation through repeated injection (four cycles). The average weight of mice injected with HmA@GCI showed an upward trend during 24 days, consistent with healthy mice (Figure 6c), and the water consumption of mice injected with HmA@GCI was reduced notably (Figure 6d). Moreover, the related indices of the whole blood were comprehensively analyzed by cytometry. As is shown in Figure 6e–g, there was no significant difference among mice, and the values were all within the normal range.

Liver is one of the most important organs of the body, which has the effect of excretion and detoxification, and it is easy to cause liver injury after exogenous drug administration. To evaluate the potential impact of HmA@GCI on liver, the level of glutamic oxaloacetic transaminase (AST), a liver function marker, in serum was measured. As shown in Figure 6h, compared with the control group, HmA@GCI‐treated mice exhibited lower levels of AST, indicating no obvious liver injury after repeated injection of HmA@GCI. Furthermore, nephropathy is one of the most important complications of diabetes and the second cause of end‐stage kidney disease. Therefore, the level of urea nitrogen (BUN), a marker of renal function, was measured (Figure 6i) to evaluate the effect of Hm@GCI on the kidney. Compared with the control group, the BUN level of diabetic mice in NS group increased significantly, while that in HmA@GCI group remained at a low level. That indicates that HmA@GCI has a good protective effect on renal function, which is mainly attributed to the good maintenance of blood glucose by the application of HmA@GCI.

Additionally, to investigate whether HmA@GCI induced an immune response, the levels of interleukin‐1*β* (IL‐1*β*) and tumor necrosis factor‐*α* (TNF‐*α*) in the serum were analyzed using enzyme‐linked immunosorbent assay (ELISA). The results demonstrated that the levels of IL‐1*β* and TNF‐*α* of HmA@GCI groups were comparable to those of healthy mice, indicating that HmA@GCI would not cause obvious immune rejection (Figure 6j,k). What's more, no obvious pathological changes in any organ were found in any mouse on the 24^th^ day (Figure 6l). The above results prove that there is no obvious toxicity to the tissues and organs, indicating that HmA@GCI is a safe and long‐lasting hypoglycemic formulation and has a good clinical application prospect. While the extreme long‐term toxicity of hexahistidine remains uncertain,^[^ ^44^, ^45^ ^]^ our study found no evidence of significant damage caused by our nanoparticle over a period of 24 days. Further research may be needed to fully understand the potential risks associated with prolonged exposure to hexahistidine.

## 3 Conclusions

In summary, we developed a pH‐responsive hexa‐histidine metal assembly (HmA) with enhanced biocatalytic cascades for glucose‐responsive Ins delivery, into which GOx, CAT, and Ins were efficiently in situ encapsulated under mild conditions. Besides protecting encapsulated proteins from protease damage inside the HmA, the biocatalytic activity of encapsulated enzymes (GOx and CAT) and the cascade reaction between GOx and CAT are enhanced, which resulted in a fast response to the variation of LBG accompanied by insulin release and efficient scavenging of noxious byproducts by GOx (H_2_O_2_). HmA@GCI reduced the LBG of diabetic mice to normal in half an hour and maintained for more than 5 days by a single subcutaneous injection, and nearly 24 days by four consecutive injections (6 days per injection). In addition, no hypoglycemia and no toxicity to tissues and organs were observed in the tested period (24 days), suggesting that HmA@GCI is a safe and long‐acting hypoglycemic agent with good clinical potential.

## 4 Experimental Section

### Materials

Zinc nitrate hexahydrate (Zn(NO_3_)_2_·6H_2_O, ≥ 99%), polyvinylpyrrolidone (PVP, Mw ≈58 k), 4‐(2‐hydroxyethyl)‐1‐piperazineethanesulfonic acid (HEPES), streptozotocin (STZ), and hydrogen peroxide solution (H_2_O_2_) were purchased from Aladdin (China). His_6_ was purchased from KareBay Biochem, Inc. and used without further purification. Ins and CAT were purchased from ShanghaiYuanye Bio‐Technology Co., Ltd. GOx was purchased from Beijing Solarbio Science and Technology Co., Ltd. FITC‐insulin (F‐Ins) was purchased from Xi'an ruixi Biological Technology Co., Ltd. The NIH3T3 cell lines and DC lines were provided by ATCC. All materials for cell culture were purchased from GIBCO. Cell Counting Kit‐8 (CCK‐8) reagent was purchased from Dojindo (Japan). Mouse IL‐1*β* and TNF‐*α* ELISA Kit were purchased from Multisciences.

### Synthesis of HmA, HmA@Ins, HmA@GI, HmA@GC, and HmA@GCI Nanoparticles

The synthesis of nanoparticles was performed according to previous reports.^[^ ^38^ ^]^ Under sonication, an aqueous solution of Zn(NO_3_)_2_·6H_2_O (0.1 m) was added dropwise to the solution containing His_6_ (4 mg mL^−1^)_,_ GOx (0.4 mg mL^−1^), CAT (0.4 mg mL^−1^), and Ins (0.4 mg mL^−1^). Then, the solution turned to light blue and was further sonicated for 10 min. Subsequently, the HmA@GCI nanoparticles were collected by centrifugation (12000 rpm, 10 min) and washed three times with deionized water. HmA@Ins, HmA@GI, and HmA@GC nanoparticles were synthesized following the same protocol by adding corresponding proteins.

### Encapsulation Efficiency of HmA@GCI Nanoparticles

EE% was calculated using F‐Ins by a microplate reader and the EE% of GOx and CAT was measured by an ultrahigh performance liquid chromatography (UPLC) system according to the following formula:

(1) $$EE\%\;=\frac{C_{1}\times \;V_{1}-\;C_{2}\times \;V_{2}}{C_{1}\;\times \;V_{1}}\;\;\times \;100\%$$

where *C*_1_ and *C*_2_ represent the concentration of the protein in the original and supernatant solutions, respectively, and *V*_1_ and *V*_2_ stand for the volume of the protein in the original and supernatant solutions, respectively.

### Glucose‐Responsive Profiles of HmA, HmA@Ins, and HmA@GCI Nanoparticles

Before testing the release of Ins, the glucose‐responsive variations in pH and particle size were tested in a 4 mg mL^−1^ glucose solution. HmA@Ins and HmA@GCI particles were redispersed into 1 mL glucose solution (4 mg mL^−1^), and shaken at 200 rpm at 37 °C. At predetermined times (0, 2, and 6 h), these particle‐containing solutions were lasered and photographed. The pH values were recorded with accurate pH test paper and the particle sizes were characterized by dynamic light scattering.

The accumulative release of Ins was tested using the above protocol by incubating HmA@Ins and HmA@GCI particles in glucose solution with different concentrations (0, 1, 4 mg mL^−1^), and F‐Ins was used instead of Ins. At the desired time points (0, 0.5, 1, 1.5, 2, 3, 4, 5, 6 h), the supernatant was collected after centrifugation (12 000 rpm, 10 min). The pellets were dispersed into another 1 mL of fresh glucose solution for further incubation. Fluorescence intensity (fl.) was recorded by a full‐wavelength microhole reader at 520 nm with excitation at 485 nm. The concentration of released F‐Ins in the supernatant was checked by the standard curve (F‐Ins concentration vs. fl.), and the cumulative release of Ins was calculated by the following formula: *C*_t_ = *C*_0_ + … + *C*_t_, where C_t_ represents the cumulative release concentration of F‐Ins at the desired time (h).

### Bioactivities of Ins, GOx, and CAT

HmA encapsulated with proteins, such as HmA@Ins, HmA@GOx, and HmA@CAT, were dispersed into PBS (pH = 4) for completely disintegrating the particles. After dialysis against with a dialysis tube (Mw = 3.5 kDa) for 2 days under 4 °C, the circular dichroism spectra of the original protein and the dialyzed proteins were recorded and secondary conformations were analyzed using the affiliated software (CDNN).

Free Ins and HmA@Ins nanoparticles (Ins: 0.4 mg mL^−1^) were co‐incubated with protease K for 2 h, and Zn^2+^ in HmA@Ins was complexed with ethylenediamine tetraacetic acid (EDTA) to release Ins. The HmA@Ins solution was dialyzed thoroughly (Mw = 500 Da). The activity of Ins was determined using an insulin ELISA in comparison with the activity of native Ins solution. Similarly, after incubating free GOx+CAT solution (Free GC) and HmA@GC nanoparticles with protease K for 2 h, the Zn^2+^ in HmA@GC was complexed according to the above steps and dialyzed, and the concentration of H_2_O_2_ was detected by H_2_O_2_ kit.

### Biocatalytic Cascades of HmA@GCI

HmA@GCI nanoparticles and Free GCI solution were dispersed into 1 mL of glucose solution (4 mg mL^−1^), respectively, and the solution was incubated at 37 °C. At preset times (5, 10, 20, 30, 40, 50, 60, 90, 120, and 180 min), the supernatant of each group was removed and the residual H_2_O_2_ in the solution was detected by H_2_O_2_ kit.

### Biocompatibility Analysis

The biocompatibility of HmA@GCI was monitored by a hemolysis assay and a CCK‐8 cytotoxicity assay. In the hemolysis experiment, HmA@Ins and HmA@GCI at different concentrations (0, 25, 50, and 100 µg mL^−1^) were mixed with 10% red blood cells (v/v) of 500 µL and incubated at 37 °C for 4 h. Pure water and red blood cells were mixed as a 100% hemolysis reference. The supernatant of the sample was centrifuged at 3000 rpm for 5 min. The absorbance at 540 nm was determined by a UV–Vis spectrometer to calculate the hemolysis rate.

For the CCK‐8 cytotoxicity assay, DC and NIH3T3 cells were seeded in 96‐well plates at a density of 1 × 10^4^ cells per well and incubated for 24 h. The culture medium was removed, and the cells were washed three times with PBS buffer. Then, HmA suspended in 1640 or DMEM at various concentrations (20, 40, 60, 80,100 µg mL^−1^) was added and incubated at 37 °C in a 5% CO_2_ atmosphere. After 24 h of incubation, the culture medium was removed and the cells were washed three times with PBS buffer. Finally, the media were replaced with 100 µL of fresh media containing 10 µL of CCK‐8 solution, and the plates were incubated for 2 h. Then, the plates were read at 450 nm using a microplate reader and five replicates were conducted for each point.

### Construction of T1D Induced by Streptozotocin (STZ)

The T1D animal model was induced by intraperitoneal injection of STZ (50 mg kg^−1^) into overnight fasted male mice for five consecutive days, followed by normal chow for another 7 days. The concentration of blood glucose was recorded daily, and the model was established successfully when the blood glucose concentration was stable above 200 mg dL^−1^ for the next 3 days. All animal procedures were performed in accordance with the Guidelines for Care and Use of Laboratory Animals of Wenzhou Medical University and approved by the Animal Ethics Committee of Wenzhou Medical University (SYXK 2015‐009).

### In Vivo Imaging

For in vivo imaging, HmA@GOx&CAT&F‐Ins was subcutaneously injected into T1D mice (200 µL, an equivalent Ins of 10 mg kg^−1^). At preset time‐points (0, 24, 48, 72, 120, and 168 h), the mice were anesthetized and imaged using a multimode optical in vivo imaging system. The qualitative analysis of florescence was carried out with the assistance of accessary software by circling the florescent area.

### In Vivo Antidiabetic Effect

The hypoglycemic effect of the nanoparticles in vivo was evaluated by monitoring the changes in blood glucose levels in T1D mice. Diabetic mice with a blood glucose level of approximately 550 mg dL^−1^ were chosen and subcutaneously injected with NS, free Ins, HmA@Ins, or HmA@GCI (an equivalent Ins of 10 mg kg^−1^), respectively. Then, the blood glucose levels were measured by a Roche blood glucose meter at the indicated time. And we also evaluated the long‐term effect of four repeated injections of HmA@GCI over a period of 4 weeks. Additionally, heathy mice were subcutaneously injected with NS, free Ins, HmA@Ins, and HmA@GCI to evaluate hypoglycemia index. Within the next 8 h after injection, the blood glucose levels were recorded every 0.5 h, and the hypoglycemia index was calculated according to the following formula.

(2) $$\mathrm{Hypoglycemia}\;\mathrm{index}\;=\frac{{\mathrm{BGL}}_{0}\;-{\;\mathrm{BGL}}_{1}}{T}$$

BGL_0_: initial LBG in mice; BGL_1_: the lowest LBG in mice; T: the time to reach the lowest blood glucose after Ins injection.***

IPGTT was used to evaluate the dynamic changes of different preparations of insulin in vivo. After fasted, healthy mice and diabetic mice were subcutaneously injected with saline, free insulin and HmA@GCI, respectively, to monitor the blood glucose levels. When the blood glucose level returned to normal, the mice were intraperitoneally injected of glucose solution (2 g kg^−1^) and we continued to monitor the blood glucose level. Finally, the time‐blood glucose level curve was drawn, and AUC was calculated.

### In Vivo Safety Evaluation

To evaluate the biocompatibility of the nanoparticles, NS and HmA@GCI were administered to mice from each group every week for four times, and healthy mice as a control group. The weight and water consumption of the mice in each group were recorded for 4 weeks. To estimate the potential toxicity of HmA@GCI, blood samples were collected on the last day of testing for red blood cell counts, white blood cell counts, platelet counts, and blood biochemical indexes, such as AST and BUN. In addition, to test the immunoreactivity, the serum of each group was sampled on the last day of testing to measure the levels of IL‐1*β* and TNF‐*α* by ELISA. Moreover, to investigate the tissue lesions, the heart, liver, spleen, lung, kidney, and skin of injection site were immediately harvested to perform by H&E staining.

### Instruments and Characterization

The sizes and zeta‐potential of the particles were measured by a Zetasizer Nano ZS instrument (Malvern, UK). The morphologies of the particles were detected by a transmission electron microscope (FEI Talos F200S microscope, USA). The circular dichromatic curves of the original Ins before encapsulation into HmA and release of Ins from HmA were recorded by a circular dichromatic spectrometer (Chirascan Plus, UK), and their secondary structures were analyzed by accessary software. A microplate reader (Varioskan LUX Multimode; Thermo, USA) was used to quantitatively analyze the absorbance of CCK‐8‐containing medium. Fluorescence spectra of F‐Ins were monitored by a full wavelength microplate reader (Thermo Fisher, USA). UPLC (Waters, USA) was used to test the concentration of proteins. Imaging of F‐Ins in vivo was performed with a Multimode optical in vivo imaging system (MOIS) (PerkinElmer, USA). Murine routine was examined using animal blood cell analyzer (Mindray, BC‐5000 vet, China). AST and BUN were detected by Fully automatic dry biochemical analyzer (IDEXX, Catalyst One, USA). HE stained sections of organs were imaged by fluorescence microscopy (DMi8 Leica, Germany).

### Statistical Analysis

All quantitative data are expressed as the mean ± standard error (SD). The significant differences between groups were determined using one‐way ANOVA followed by Tukey's test (GraphPad Prism version 5). A statistically significant difference was considered at a minimal level of significance of *p* < 0.05, and denoted as ^*^ *p* < 0.05, ^**^ *p* < 0.01, ^***^ *p* < 0.001, ^****^ *p* < 0.0001.

## Conflict of Interest

The authors declare no conflict of interest.

## Supporting information

Supporting Information

## Data Availability

The data that support the findings of this study are available from the corresponding author upon reasonable request.
