# Supplementary material for: pH‐Responsive Hexa‐Histidine Metal Assembly (HmA) with Enhanced Biocatalytic Cascades as the Vehicle for Glucose‐Mediated Long‐Acting Insulin Delivery
Source: Adv Sci (Weinh). 2023 Jun 2;10(23):2301771. doi: 10.1002/advs.202301771 (PMC10427356; doi:10.1002/advs.202301771)
Supplement: Supplementary file 1 — Supporting Information [file ADVS-10-2301771-s001.pdf]

ADVANCED SCIENCE
Open Access

Supporting Information

for *Adv. Sci.*, DOI 10.1002/advs.202301771

pH-Responsive Hexa-Histidine Metal Assembly (HmA) with Enhanced Biocatalytic Cascades as the Vehicle for Glucose-Mediated Long-Acting Insulin Delivery

Sijie Zhou, Ruhui Yang, Xiaoling Xie, Liwen Wang, Shengwu Zheng, Na Li, Sicheng Tang* and Xingjie Zan**

Supporting Information

pH-Responsive Hexa-histidine Metal Assembly (HmA) with Enhanced Biocatalytic Cascades as the Vehicle for Glucose-Mediated Long-acting Insulin Delivery

Sijie Zhou, Ruhui Yang, Xiaoling Xie, Liwen Wang, Shengwu Zheng, Na Li*, Sicheng Tang* and Xingjie Zan*

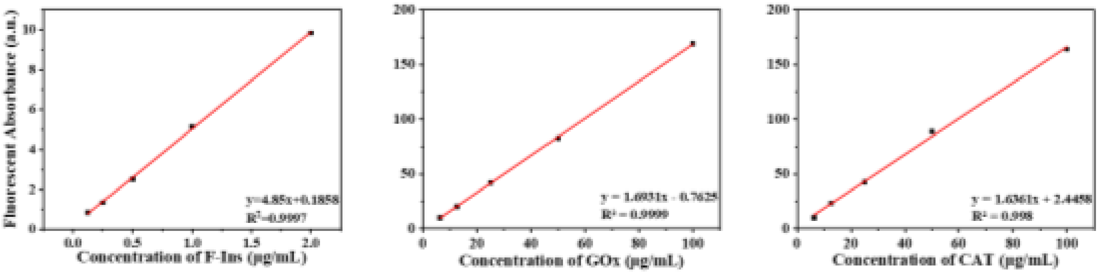

Figure S1. The working curves of F-Ins, GOx and CAT.

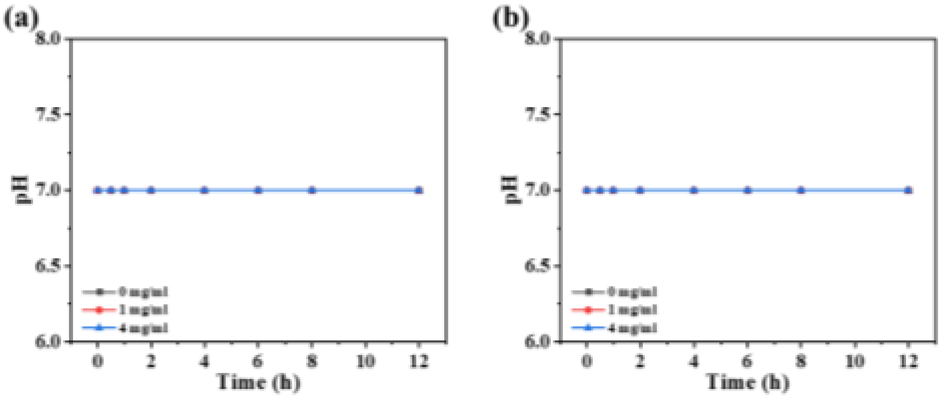

Figure S2. pH changes with the incubation time of a) HmA and b) HmA@Ins particles into glucose solution with concentration of 0, 1 and 4 mg/mL.

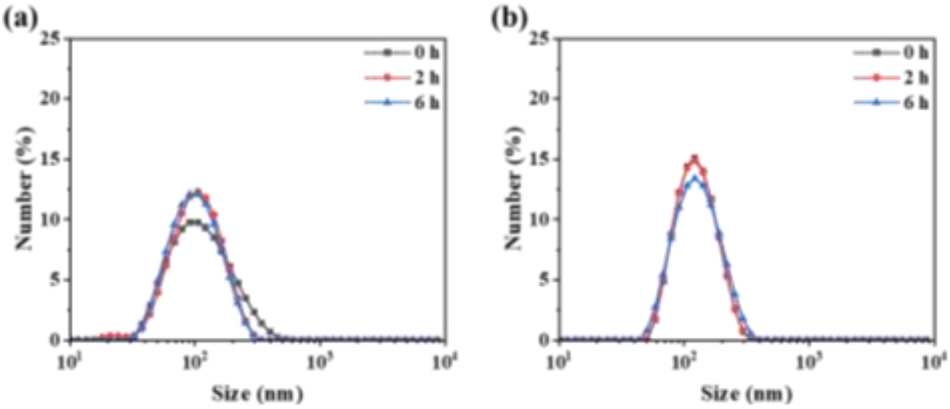

Figure S3. The size change of a) HmA and b) HmA@Ins after incubating with glucose solution (4 mg/mL) at different time points.

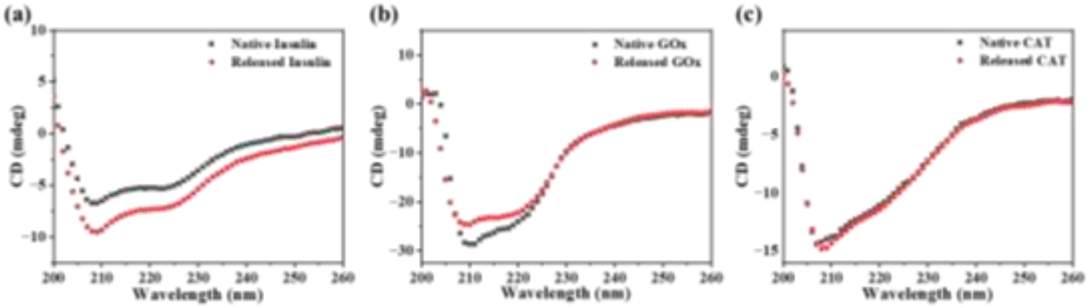

Figure S4. The CD spectra of native and released a) Ins, b) GOx and c) CAT from HmA@Ins, HmA@GOx and HmA@CAT, respectively.

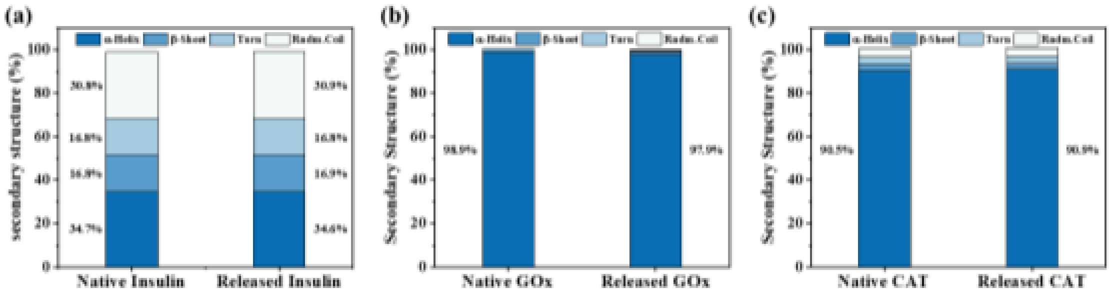

Figure S5. The secondary structure of native and released a) Ins, b) GOx and c) CAT from HmA@Ins, HmA@GOx and HmA@CAT, respectively.

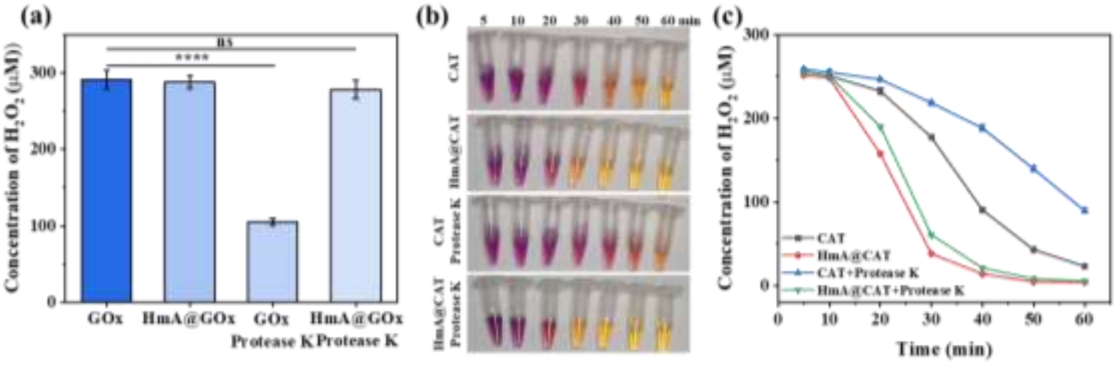

Figure S6. a) Bioactivity assays of HmA@GOx and their native counterparts with/without protease K treatment. b) Visual color change in the FOX solution after adding the mixed solution of H_2O_2 and samples (free catalase and HmA@CAT with proteinase K (bottom two panels) and without proteinase K (top two panels)) with reaction time. Fast color change means high bioactivity. c) Kinetics of the above samples to degrade H_2O_2.

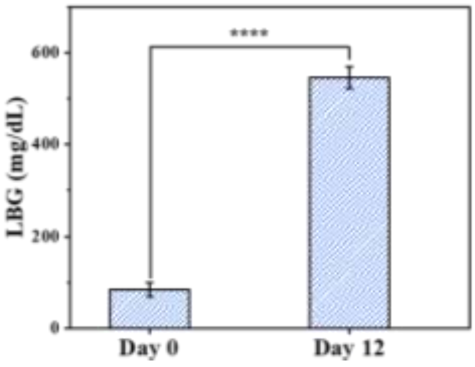

Figure S7. LBG in mice before and after modeling.
